# High Prevalence and Endemicity of Multidrug Resistant* Acinetobacter* spp. in Intensive Care Unit of a Tertiary Care Hospital, Varanasi, India

**DOI:** 10.1155/2018/9129083

**Published:** 2018-07-02

**Authors:** Tuhina Banerjee, Anwita Mishra, Arghya Das, Swati Sharma, Hiranmay Barman, Ghanshyam Yadav

**Affiliations:** ^1^Department of Microbiology, Institute of Medical Sciences, Banaras Hindu University, Varanasi 221005, India; ^2^Department of Anaesthesiology, Institute of Medical Sciences, Banaras Hindu University, Varanasi 221005, India

## Abstract

The increasing emergence of* Acinetobacter *spp. with healthcare associated infections (HCAI) in intensive care units (ICU) is alarming. This study was a laboratory-based audit to determine the prevalence of* Acinetobacter *spp. associated with HCAI in the adult ICU of a tertiary care hospital in Varanasi, north India, with special reference to antimicrobial resistance and resistance determinants over a period of 5 years. A total of 993 cases of HCAI were analyzed. Isolates were characterized as multidrug resistance and extended drug resistance (MDR/XDR) based on antimicrobial susceptibility records. Few (100) randomly selected isolates of* Acinetobacter baumannii* (*A. baumannii)* were tested for imipenem, meropenem, and polymyxin B susceptibility by minimum inhibitory concentration (MIC) and for the presence of class A and B carbapenemases by multiplex PCR. Active surveillance of ICU environment was also performed. High prevalence of* Acinetobacter* related hospital acquired pneumonia (HAP) with significant resistance to imipenem (p<0.05) and 88.02% MDR and 61.97% XDR was detected along with persistence in the ICU environment. The isolates harbored* bla*_*IMP*_ (89%),* bla*_*VIM*_ (51%), *bla*_*NDM*-1_ (34%), and *bla*_OXA-23-like_ (93%) genes. Specific interventional measures should be adopted to control these imipenem resistant* Acinetobacter* spp. which have attained the level of endemicity in our ICU setup.

## 1. Introduction

The intensive care unit (ICU) in a hospital is a unique setting having both patients with compromised immune status and conditions conducive to the growth of microorganisms. On one hand it houses critically ill patients, while at the same time it also provides a suitable environment for proliferation and persistence of several multidrug resistant organisms (MDROs) amidst high antibiotic pressure [1]. Several factors like over the counter antibiotic use, overcrowding in hospitals, imperfect infection control practices, and use of excessive invasive devices contribute to the development of high antimicrobial resistance, especially in developing countries [2]. Additionally, these factors also facilitate easy transmission of MDROs implicated in various healthcare associated infections (HCAI). One such MDRO that has rapidly reached the level of a ‘significant pathogen' from a commensal of ‘little significance' is* Acinetobacter* spp. [3]. The tremendous ability of this organism to accumulate antibiotic resistant determinants in response to antibiotic challenges and resist adverse conditions causing initial colonization and subsequent infection is really bothersome [4]. There has been recent emergence of* Acinetobacter* spp. in both developing and developed countries revealing its potential to cause sustained outbreaks within the ICU and resilience to the nosocomial environment [5]. With worldwide reports of increasing isolation of this organism from the ICU, we performed a laboratory-based audit of HCAI with special reference to* Acinetobacter* spp. to estimate the extent of the problem in the adult ICU of the tertiary care hospital and also analyze the prevalent situation for possible control measures.

## 2. Material and Methods

### 2.1. Study Site and Design

This was a laboratory-based study conducted in the Department of Microbiology and the 25 bedded adult ICU of the associated 1200 bedded tertiary care university hospital in Varanasi, north India. The study involved collection, classification, and analysis of data retrospectively over a period of 5 years (January 2012 to December 2016) with special reference to* Acinetobacter* spp. followed by characterization of the collected representative* Acinetobacter* isolates in a prospective manner (January to September 2017). As the laboratory-based passive surveillance was performed as a part of the routine management programme of the Infection Control Team, it was approved by the hospital infection control committee. Further microbiological study of the collected isolates was approved by the Institute ethical committee (No. 2018/EC/321).

### 2.2. Definitions and Source of Isolates

The study involved classification of infections based on reference definitions as given below. Healthcare associated infection (HCAI) was referred to as infections acquired during the process of patient care in the hospital or healthcare facility and was not present or incubating at the time of admission [6]. Only those cases with clinical suspicion of HCAI that had developed after 48 hours of admission in the ICU were considered. Hospital acquired pneumonia (HAP) and ventilator associated pneumonia (VAP) were categorized based on clinical, laboratory, radiological, and microbiological data according to the Clinical Pulmonary Infection Score (CPIS) [7]. For blood stream infections (BSI), only microbiologically confirmed cases of clinical sepsis were considered in patients with and without intravascular devices [8]. Surgical site infections (SSI) were broadly considered as cases with evidence of infection following surgery either from the incision or drainage site or following any organ involvement [8]. Relevant samples, namely, endotracheal aspirate/secretions, bronchoalveolar lavage, pleural fluid, and sputum, were considered for HAP, conventional blood culture and/or central venous catheter tip culture, cerebrospinal fluid (CSF) for BSI, and pus or body fluids following surgery for SSI. As very few cases of Catheter associated urinary tract infections (CAUTI) were involved in this period with inconclusive details, we did not involve cases of UTI in this study. Data from those cases were considered where relevant details of the HCAI were present. Cases with ambiguous diagnosis, microbiological data suggesting colonization, and polymicrobial flora consisting of more than 2 organisms were excluded. Only one sample per patient for a specific HCAI was considered for the study.

### 2.3. Isolation and Identification of Acinetobacter Isolates from the ICU Environment

During the study period, surface swab samples from bedrails and beddings, humidifiers, ventilation masks, instruments and probes in vicinity of the patients, stethoscope, fluid sets, door handles, suction tubes, and dressing trolley were collected at repeated intervals randomly for microbiological surveillance of the ICU environment. Hand samples of healthcare personnel in the ICU, handwash, handrubs, and antiseptics were also surveyed. Further processing followed inoculation and culture of the samples on blood agar and MacConkey agar (Hi Media, Mumbai, India) media. Isolates were identified by standard biochemical tests [9]. Similarly, major operation theatres of the hospital were also surveyed as a part of infection control procedures.

### 2.4. Antimicrobial Susceptibility Data

Records of antibiotic susceptibility testing by modified Kirby Bauer disc diffusion method according to CLSI (2017) [10] were analyzed with the following discs, namely, for nonfermenters like* Acinetobacter* and* Pseudomonas* spp. piperacillin (100*μ*g), piperacillin/tazobactam (100/10 *μ*g), ceftazidime (30*μ*g), ceftriaxone (30 *μ*g), cefepime (30 *μ*g), imipenem (10 *μ*g) and meropenem (10 *μ*g), gentamicin (10 *μ*g), amikacin (30 *μ*g), ciprofloxacin (5 *μ*g), levofloxacin (5 *μ*g), and cotrimoxazole (1.25/23.75 *μ*g). Isolates were classified as multidrug resistant (MDR) and extensively MDR (XDR) as per reference. Briefly, the isolates showing resistance to ≥1 antimicrobial agents in ≥3 antimicrobial categories were considered as MDR (multidrug resistant) and resistance to ≥1 antimicrobial agent in all but ≤ 2 antimicrobial categories was included as XDR (extensively drug resistant) [11].

### 2.5. Determination of MIC for Acinetobacter Isolates

Further from the total MDR* Acinetobacter* spp. isolated during the study period, 100 isolates of* Acinetobacter baumannii* (*A. baumannii) *were randomly chosen and subjected to determination of minimal inhibitory concentrations (MIC) for imipenem (Lupin Ltd., Mumbai, India), meropenem (Aristo Pharmaceuticals, Mumbai, India), and polymyxin B (Sigma, USA) by agar dilution method (CLSI, 2017) [10]. For susceptibility testing,* Escherichia coli* ATCC 25922 and* Pseudomonas aeruginosa* ATCC 27853 were used as control.

### 2.6. Molecular Characterization of A. baumannii with Reference to Carbapenemases

All the 100 MDR isolates were confirmed for* A. baumannii* by the presence of *bla*_OXA-51-like_ as per [12]. Further they were characterized for presence of carbapenemase genes. Presence of class A carbapenemase determinants (GES, IMI/NMC-A, SME, and KPC) [13] and class B carbapenemase determinants (IMP, VIM, OXA-48, and NDM-1) [14] were also looked for by multiplex PCR as per previous protocol without any modification. High level carbapenem resistance in these isolates was detected by the presence of *bla*_OXA-23-like_ [12].

### 2.7. Data Analysis

All relevant data from the cases included in the study were tabulated in Microsoft Excel 2010. Prevalence rates and antibiotic resistance profile were computed and infections due to* Acinetobacter* were compared with infections due to other microbial causes by Fisher's exact test. All statistical analysis was performed by MedCalc Statistical Software version 16.4.3 (Ostend, Belgium).

## 3. Results

### 3.1. Samples and Cases

In this study, a total of 2984 samples from same number of patients were considered with clinical evidence of HCAI. Among these, 993 (33.68%) samples yielded positive growth (Table 1) and were identified as pathogens. Male patients predominated over female counterparts in the ratio 1.7:1 (1861/1087). Mean age of the patients was 39±4.7 years. Of the 993 cases of suspected HCAI, data regarding antibiotics used empirically were present only in 415 cases, in which there were 84.09% (349/415), 69.63% (289/415), 57.83% (240/415), and 65.3% (271/415) use of imipenem, piperacillin-tazobactam, third generation cephalosporins, and fluoroquinolones, respectively.

### 3.2. Prevalence of HCAI and Microbial Etiology

**Figure 1:**
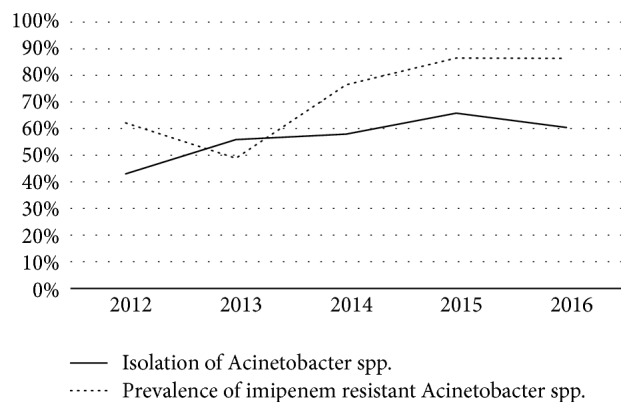
Prevalence of* Acinetobacter* spp. and imipenem resistance in the study period.

Among the suspected cases of HCAI, HAP (23.39%) was the most common infection followed by BSI (7.5%) and SSI (2.37%) (Table 2). Of the total HAP, 683 cases (97.85%) were classified as VAP while of the total BSI, 30 (13.39%) cases were associated with central line catheters. Additionally, culture positivity was significantly more in samples from patients with suspected HAP than other infections (p<0.0001). Considering overall samples, Gram-negative bacilli were dominant (84.49%, 839/933) as compared to Gram positive organisms and fungi.* Acinetobacter* spp. (42.9%) were the most common organism isolated followed by* Klebsiella* spp. (15.10%),* E.coli* (11.17%), and* Pseudomonas* spp. (10.17%). In case of HAP,* Acinetobacter* was the most significant organism associated with the condition as compared to other Gram-negative bacilli (p<0.001). Similarly, members of* Enterobacteriaceae* family were significantly associated with SSI as compared to nonfermenters (p<0.001). There was no significant change in prevalence of infections by* Acinetobacter* spp. over the years as shown in Figure 1. Majority of the* Acinetobacter* isolates (314/426, 96.94%) were biochemically presumed as* A. baumannii*.

### 3.3. Antimicrobial Resistance Profile

Among the nonfermenters, considerable resistance was seen with almost all the antibiotics tested as shown in Table 3. For* Acinetobacter* spp., majority of the antibiotics were ineffective with resistance rates varying from 76.99% to 92.01%. However, imipenem showed nearly 30% susceptibility for these isolates. Additionally, imipenem resistance in* Acinetobacter* spp. was significantly associated with those strains that were isolated from HAP cases as compared to other HCAI (p=0.005). For* Pseudomonas* spp., resistance rates were lower than* Acinetobacter* spp., particularly for piperacillin-tazobactam and imipenem. Among the* Acinetobacter* isolates, 88.02% (375) were MDR while 61.97% (264) were XDR. There was increase in imipenem resistant MDR isolates since 2013 following a decrease in the past one year (Figure 1).

All the 100 MDR isolates were imipenem resistant* A. baumannii *which showed MIC values of >32*μ*g/ml for both imipenem and meropenem among which 75% isolates had MIC > 128*μ*g/ml. All the isolates were susceptible to polymyxin B (MIC ≤ 2*μ*g/ml).

### 3.4. Molecular Characterization of A. baumannii Isolates

**Figure 2:**
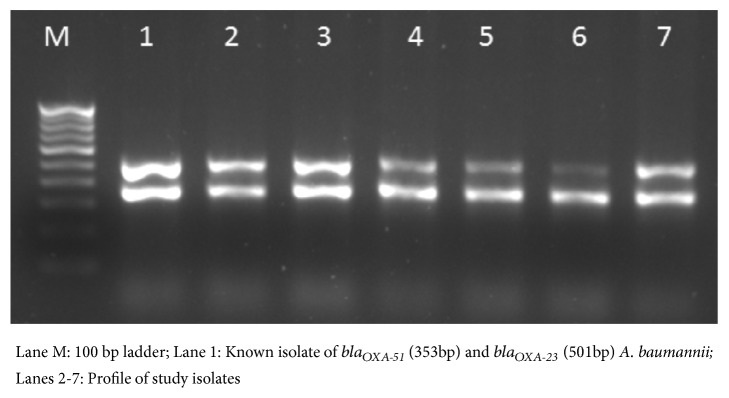
Amplification of *bla*_*OXA*-51_ and *bla*_*OXA*-23_ by multiplex PCR.

**Figure 3:**
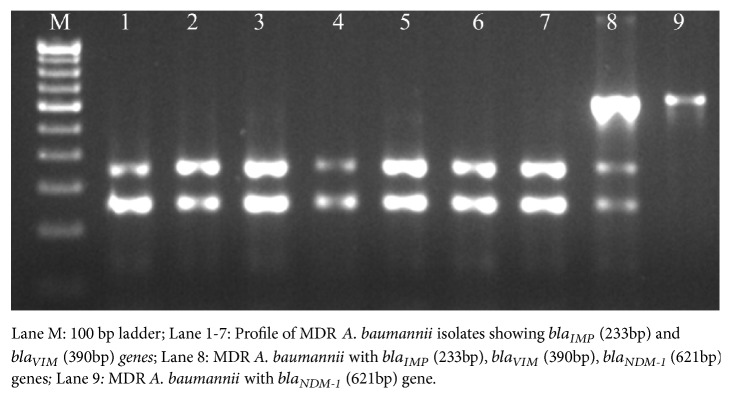
Amplification of class B carbapenemase genes by multiplex PCR.

Among the 100 MDR isolates of* A. baumannii* confirmed by presence of *bla*_OXA-51-like_ gene, 93 isolates (93%) were associated with *bla*_OXA-23-like_ gene as shown in Figure 2. Among the carbapenemases, class A carbapenemases were not detected in any of the isolates. However, class B carbapenemases in the frequency of 89% for *bla*_IMP_, 51% for *bla*_VIM_, and 34% for *bla*_NDM-1_ were found as shown in Figure 3.

### 3.5. Environmental Surveillance

During this period, on few occasions* Acinetobacter* spp. were isolated from bedrails, surfaces of monitors, door handles, and wash basins. However, the organism was never isolated from hands of any healthcare personnel in the ICU. Of these, 63.63% (14 out of 22) of the isolates were imipenem resistant with 27.27% (6 out of 22) harboring the *bla*_*NDM*-1_ gene.

## 4. Discussion

The recent increase in worldwide reports of* Acinetobacter *spp. and antimicrobial resistance associated with it especially in the nosocomial setup has raised an alarm among the clinicians and microbiologists. In line with the situations in other ICUs, the study showed the extent of the problem of this ‘once opportunistic and now established pathogen' in our adult ICU of the tertiary care hospital that serves as a referral centre for many hospitals in and around Varanasi in north India.

Incidence of infections in the ICU usually ranges from 2.3% to 49.2% as evident from available literature, with variations depending on the type of population studied in a particular setting [15]. The present study documented a high prevalence of healthcare associated infections of 33.68% based on laboratory data. One of the recent studies from adjoining country of Nepal has also reported comparable ICU infections rate of 27.4% [16] while even higher rates of nearly 51% have been reported in previous studies [17]. In Indian setup, nosocomial infection incidence rates of 11.97% and 17.7% have been reported in the recent past in the ICUs of tertiary care hospitals [15, 18]. As rates of nosocomial infections are dependent on the local epidemiology and hospital conditions, the spectrum of microbial etiologies also differ. While Gram-negative organisms are the most prevalent causes of infections in developing countries, Gram positive organisms usually predominate in developed countries in the West [19]. Unregulated use of antibiotics in developing countries as compared to West has often been implicated as a major reason responsible for this difference in epidemiology [19].

Among the different HCAIs, pneumonia and BSI are the most complicated infections accounting for mortality [1]. In the ICU, majority of these cases of pneumonia acquired after hospital admission are attributed to the use of mechanical ventilation. It has been speculated that nearly 50% of HAP are VAP [1]. However, this study shows even higher prevalence of VAP among HAP (683/697). Moreover it also revealed that* Acinetobacter *spp. were the significant major pathogen against other Gram-negative organisms. Global reports suggest that it is* Acinetobacter *spp. as the only Gram-negative bacilli to have increased significantly as a causative agent of VAP as documented in ICUs of developed countries over 15 years [20]. High prevalence of* Acinetobacter* spp. from HAP has also been reported from other Asian countries [16, 21] as well as from the Indian subcontinent where not only HAP, but* Acinetobacter* was the commonest isolate in the ICU from CAUTI and CLABSI and other device associated infections [22]. This study also showed* Acinetobacter* spp. as the predominant pathogen isolated from blood culture. On the contrary, in ICUs of Spain and Western Canada [1, 23],* Acinetobacter* spp. were not among the major pathogens in nosocomial bacteremia. BSI is most commonly associated with indwelling central venous catheters but may also be caused due to other foci of infections. In this study, majority of the BSI (86.61%) were not associated with central catheters but had other reasons of infections which could not be ascertained from the available records. It becomes imperative to use broad spectrum antibiotics empirically in cases of BSI due to life threatening situations and delayed reports when using conventional culture methods as seen in most of the setups in developing countries [1]. We found a low surgical site infection rate of 2.37% as compared to other studies among the other HAIs [16]. Additionally, in line with this finding we did not find any noncompliance with the maintenance of operation theatres and infection control practices in the surgical procedures through the microbiological surveillance performed routinely during this period.

One of the major causes of emergence of* Acinetobacter *spp. as the predominant organism in the ICUs in a short span of time is its ability to exhibit and acquire drug resistance. It has been speculated that there are hardly any alternatives to tackle these MDR* Acinetobacter* spp. other than adhering to the available strategies in a stringent manner [24]. This study demonstrated higher prevalence of drug resistance among the* Acinetobacter* isolates towards majority of the antibiotics and prevalence of 88.02% MDR and 62% XDR. Similar reports of multidrug resistant isolates have been reported with 100% resistance to all the commonly applied antibiotics except tigecycline and colistin [25]. In another retrospective audit from the subcontinent,* Acinetobacter* spp. have been studied to be the commonest isolate from respiratory site in an ICU with 63.8% infections being acquired in the ICU and MDR antibiotic profile in 70% of such isolates [26]. An increase in proportion of MDR* Acinetobacter* from 89.4% to 95.9% over a period of 5 years has been demonstrated in a study with significant proportion of these MDR isolates being isolated from respiratory samples [27]. A study from Poland over 10 years period has reported* Acinetobacter* spp. as the commonest ICU pathogen with 87% XDR and a rapid increase in carbapenem resistance over the years [28]. Our study however did not show any rapid change in the isolation of* Acinetobacter* isolates throughout the study period of 5 years but definitely demonstrated increasing emergence of imipenem resistant isolates. This could suggest that this organism has reached the level of endemicity in our ICU setup, a fact supported by isolation of* Acinetobacter* spp. from the ICU environment on several occasions during the study period denoting their persistence and survival in the hospital environment. In this context, a large surveillance study had revealed about 70% of the patients in the ICU being administered antibiotics either prophylactically or for therapeutics [17]. Similarly, one previous study from the same ICU had revealed heavy empirical antibiotic use owing to the fact that majority of the patients were being transferred from other parts of the hospital while on indiscriminate antibiotics [29]. In addition, the mentioned study also reported a low compliance with the antibiotic policy in the same setup. Interestingly, the initial decrease in imipenem resistant isolates observed in the present study could have been due to implementation of the policy though with less success which could not be sustained due to noncompliance. The present study also documented empirical use of imipenem under similar circumstances. With such high selective pressure due to intense antibiotic use, it becomes essential for the MDROs to acquire several drug resistance mechanisms as a part of evolutionary biology. Based on this, we hypothesized that empirical carbapenem (mostly imipenem) use in the ICU could have provided a survival advantage for the MDR* Acinetobacter* isolates to multiply and persist in the ICU environment and maintain their endemicity.

Of the several mechanisms of acquisition of drug resistance, production of metalloenzyme and carbapenemases has been an important strategy of survival, especially among isolates of* Acinetobacter* spp. from VAP [7]. This study showed the relative abundance of carbapenemase encoding genes 89% for *bla*_IMP_, 51% for *bla*_VIM_, and 34% for *bla*_NDM_. Among* Acinetobacter* spp., metallo-beta-lactamase (MBL) production has been reported to be as high as 42% in one of the studies with *bla*_IMP_ as the most prevalent gene [30], while another study reported the predominance of *bla*_VIM_. [31]. Multidrug resistant* Acinetobacter* spp. have been reported to be the major cause of morbidity and mortality after carbapenem resistant* Enterobacteriaceae* (CRE) and extended spectrum beta lactamases (ESBL) producing* Enterobacteriaceae *[2]. The MBL genes are often plasmid mediated and there have been reports of cross genes transfer from* Enterobacteriaceae* [32]. In similar context, the ‘oxa' family of carbapenemases has been increasingly reported especially that of *bla*_OXA-23_ from developing countries [2]. In a study from Indonesia [21], 91.8% of the* Acinetobacter* spp. in the ICU were producer of *bla*_OXA-23_ as compared to 93% in this study, which confers high level carbapenem resistance.

Lastly, this study revealed the burden and endemicity of carbapenem resistant* Acinetobacter* spp. in the adult ICU of the tertiary care hospital of north India. Despite limitations like inability to conduct Centers for Disease Control and Prevention/ National Healthcare Safety Network (CDC/NHSN) based audit of HCAI due to limitation of resources and lack of appropriate data and failure to analyze the risk factors associated with the situation, the study provides relevant data for assessment and implementation of appropriate strategies to control the situation. As microbiological data is the leading factor for deciding upon the therapy and infection control strategies, we emphasized strict implementation of antibiotic stewardship programme along with stringent infection control measures to prevent transmission and persistence of the pool of MDR* Acinetobacter* spp. from the ICU.

## 5. Conclusions

High burden of imipenem resistant* Acinetobacter* spp. harboring multiple carbapenemase encoding genes and especially associated with VAP was revealed in the adult ICU of the tertiary care hospital by this study. Endemicity of the organism in the ICU environment amidst intense antibiotic pressure seems to be the most probable cause for this situation. Stringent measures to eradicate the reservoir of MDR* Acinetobacter* spp. should be targeted by specific interventional methods for effective control.

## Figures and Tables

**Table 1:** Prevalence of HCAI in the ICU over a period of 5 years.

**Type of infection **	**Samples received **	**Samples positive by culture n (**%**)**	**Prevalence (**%**)**
Hospital acquired pneumonia (HAP)	1055	698 (66.16)	23.39

Blood stream infections (BSI)	1798	224 (12.45)	7.5

Surgical site infections (SSIs)	131	71 (54.19)	2.37

Total	2948	993 (33.68)	

**Table 2:** Distribution of causative agents isolated from various HCAI based on culture.

**Organism group**	**Members **	**HCAI**			**Total (**%**)** **n= 993**
		**HAP** **∗** ** (698) (**%**)**	**BSI (224) (**%**)**	**SSI** ^**#**^ ** (71) (**%**)**	
Gram negative bacteria Non-fermenters	*Acinetobacter *spp	363 (52.00)	49 (21.87)	14 (19.71)	426 (42.90)*∗*
	*Pseudomonas* spp.	77 (11.03)	14 (6.25)	10 (14.08)	101 (10.17)

Gram negative bacteria Enterobacteriaceae	*Klebsiella *spp	90 (12.89)	42 (18.75)	18 (25.35)	150 (15.10)^ #^
	*Escherichia coli*	78 (11.17)	16 (7.14)	17 (23.94)	111 (11.17)^ #^
	*Citrobacter* spp.	23 (3.29)	10 (4.46)	5 (7.04)	38 (3.82)^ #^
	Others	8 (1.14)	3 (1.33)	2 (2.81)	13 (1.30)^ #^

Gram positive bacteria	*Enterococcus* spp.	3 (0.42)	14 (6.25)	3 (4.22)	20 (2.01)
	*Staphylococcus* spp.	18 (2.57)	46 (20.53)	2 (2.81)	66 (6.64)

Fungi	*Candida* spp.	38 (5.44)	30 (13.39)	0	68 (6.84)

Total		698	224	71	

*∗*HAP is significantly associated with *Acinetobacter* spp., p<0.001.

^#^SSI is significantly associated with *Enterobacteriaceae.*, p<0.001.

**Table 3:** Resistance profile of non-fermenters isolated from various HCAI.

**Antimicrobial category**	**Antibiotics **	***Acinetobacter* spp.**			**Total (**%**) n=426**	***Pseudomonas* spp.**			**Total (**%**) n=101**
		**HAP (**%**) (363)**	**BSI (**%**) (49)**	**SSI (**%**) (14)**		**HAP (**%**) (77)**	**BSI (**%**) (14)**	**SSI (**%**) (10)**	
Penicillins	Piperacillin	337 (92.83)	35 (71.42)	12 (85.71)	384 (90.14)	47 (61.03)	6 (42.85)	6 (60)	59 (58.41)

Penicillins + *β*-lactamase inhibitors	Amoxicillin-clavulanate	342 (94.21)	37 (75.51)	13 (92.85)	392 (92.01)	-	-	-	-
	Piperacillin-tazobactam	314 (86.50)	31 (63.26)	9 (64.28)	354 (83.09)	32 (41.55)	4 (28.57)	3 (30)	39 (38.61)

Extended-spectrum cephalosporins; 3rd and 4th generation	Ceftazidime	343 (94.49)	37 (75.51)	12 (85.71)	392 (92.01)	59 (76.62)	7 (50)	5 (50)	71 (70.290
	Ceftriaxone	336 (92.56)	40 (81.63)	12 (85.71)	388 (91.07)	-	-	-	-
	Cefepime	337 (92.83)	35 (71.42)	11 (78.57)	383 (89.90)	52 (67.53)	8 (57.14)	6 (60)	66 (65.34)

Carbapenems	Imipenem	266*∗* (73.27)	24 (48.97)	8 (57.14)	298 (69.95)	28 (36.36)	6 (42.85)	3 (30)	37 (36.63)
	Meropenem	315 (86.77)	35 (71.42)	10 (71.42)	360 (84.50)	53 (68.83)	5 (35.71)	5 (50)	63 (62.37)

Aminoglycosides	Gentamicin	322 (88.70)	33 (67.34)	11 (78.57)	366 (85.91)	52 (67.53)	6 (42.85)	6 (60)	64 (63.36)
	Amikacin	313 (86.22)	32 (65.30)	10 (71.42)	355 (83.33)	44 (57.14)	6 (42.85)	6 (60)	56 (55.44)

Fluoroquinolones	Ciprofloxacin	336 (92.56)	33 (67.34)	9 (64.28)	378 (88.73)	59 (76.62)	3 (21.42)	6 (60)	68 (67.32)
	Levofloxacin	285 (78.51)	32 (65.30)	11 (78.57)	328 (76.99)	60 (77.92)	3 (21.42)	6 (60)	69 (68.31)

Folate pathway inhibitors	Trimethoprim sulfamethoxazole	319 (87.87)	35 (71.42)	10 (71.42)	364 (85.44)	-	-	-	-

MDR/XDR strains		MDR= 375 (88.02%) XDR=264 (61.9%)				MDR= 72 (71.28%) XDR= 58 (57.42%)			

*∗*Imipenem resistance significantly associated with HAP, p=0.005.

## Data Availability

The data used to support the findings of this study are available from the corresponding author upon request.
